# An approach to reconstructing a segmental defect of the mandible involving the condyle secondary to nodular fasciitis in a pediatric patient: a case report

**DOI:** 10.1186/s13256-025-05269-0

**Published:** 2025-05-13

**Authors:** Nitin Bhola, Shreya Pawar

**Affiliations:** Department of Oral and Maxillofacial Surgery, Sharad Pawar Dental College, Datta Meghe Institute of Higher Education and Research (DMIHER), Sawangi (M), Wardha, Maharashtra 442001 India

**Keywords:** Nodular fasciitis, Temporomandibular joint replacement, Patient-specific implant, Virtual surgical planning, Case report

## Abstract

**Background:**

Nodular fasciitis is a rare, benign, and rapidly growing soft tissue lesion that predominantly affects the fascia and is typically observed in the extremities. However, its occurrence in the head and neck region is uncommon, making it a challenging entity to diagnose and treat. In the head and neck, the lesion can involve various structures including the soft tissue, muscles, and even the parotid gland. Clinically, patients may present with swelling, pain, or a rapidly enlarging mass often leading to misdiagnosis with more malignant conditions such as sarcomas or fibromatosis.

**Case presentation:**

We present a case of a 12-year-old boy of Indian ethnicity with a progressively growing swelling over the face, which was initially thought to be a malignant tumor owing to its rapid growth. Biopsy confirmed the diagnosis of nodular fasciitis. Imaging revealed a large expansile lytic lesion involving the left hemimandible. The lesion was surgically excised and reconstruction and total joint replacement were done using patient-specific implants.

**Conclusion:**

This report highlights the importance of considering nodular fasciitis in the differential diagnosis of rapidly growing soft tissue masses in the head and neck region. Prompt recognition and surgical intervention can lead to favorable results, restoring both function and esthetics for patients affected by this benign yet challenging condition.

## Introduction

Nodular fasciitis is a rare reactive lesion, which is benign in nature, originating from fibroblasts and myofibroblasts [1]. It was first described by Konwaler in 1955 who named it as subcutaneous pseudosarcomatous fibromatosis [2]. In 1961, Stout introduced the term “nodular fasciitis” hypothesizing that the lesion originated from both the superficial and deep fascial layers [3]. The common site of occurrence is the upper extremities. However, it has also been reported at sites such as the head and neck, lower extremities, and the chest and back [4]. The age predilection for this lesion is between 20 and 50 years [5]. It is rarely seen in pediatric populations [6].

The pathophysiology of this entity is not exactly known. It is thought to be caused by unusual proliferation of myofibroblasts. The etiology of this lesion is thought to be due to trauma and gene fusion. In around 10–15% of cases, this lesion was associated with trauma. Some studies have shown that nodular fasciitis is caused by a recurrent gene fusion event involving the non-muscular myosin, *MYH9*, and ubiquitin-specific protease, *USP6*. This gene fusion event overproduces a chimeric protein that can appropriately stimulate cell signaling pathways that may promote the development of tumors [7, 8].

Nodular fasciitis presents as a rapidly growing solitary lump or a mass that is painless throughout its course. Owing to its rapid growth, high mitosis, and high cellularity, it is often perceived to be a sarcoma. It can be differentiated from the latter as it has a lesser degree of hypercellularity and mitosis, and denser myxoid cytoplasm as compared with the sarcoma [9]. Schwannoma presents as a nodular, well-circumscribed growth that is generally painful with a radiating nature. However, Schwannomas are positive for S100 protein, showing an epidermal origin, which is negative in nodular fasciitis. The tumor cells of nodular fasciitis are positive for α-SMA, muscle specific actin (HHF-35), and vimentin, which suggests a myofibroblastic differentiation [10].

We herein report a rare case involving a pediatric patient who exhibited significant swelling, leading to considerable clinical uncertainty. The condition was successfully managed through surgical intervention, leveraging cutting-edge advancements in virtual surgical planning and the use of patient-specific implants to enhance rehabilitation outcomes.

## Case presentation

**Fig. 1:**
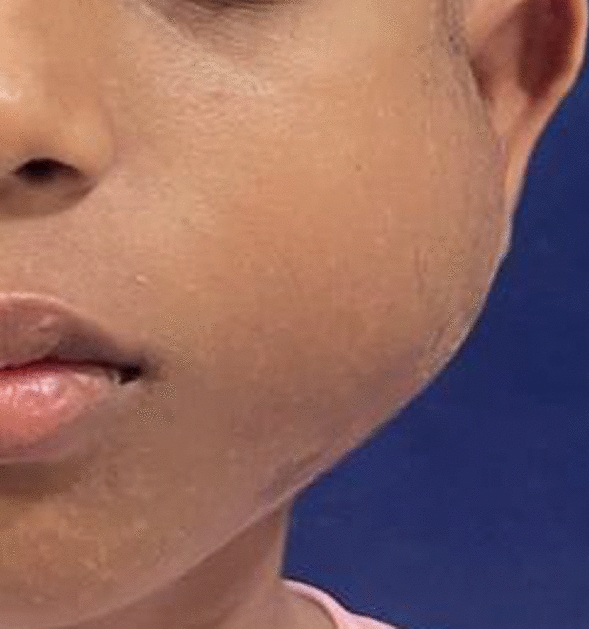
Frontal photo of the patient

A 12 year old boy of Indian ethnicity presented to the outpatient department with the presence of a single, progressively growing, firm, diffuse swelling over left side of his face, which had gradually increased over 4 months (Fig. 1).

**Fig. 2:**
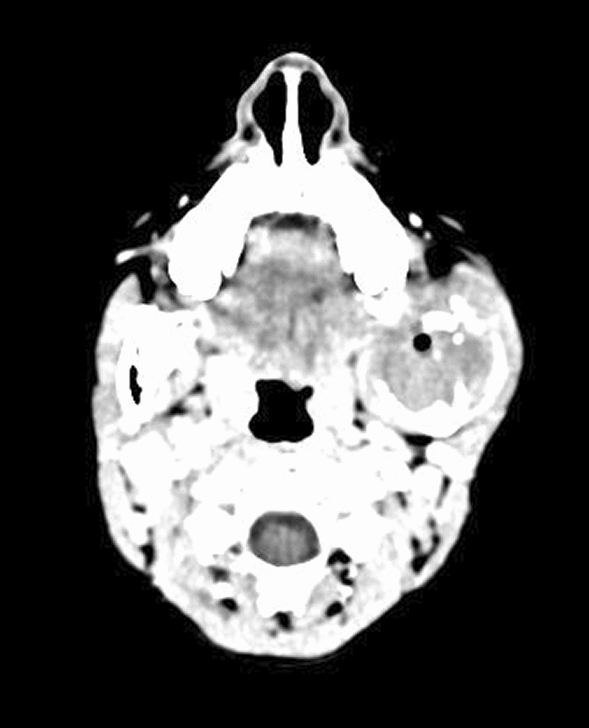
Axial section of the contrast enhanced computed tomography scan showing gross destruction of the mandible

**Fig. 3:**
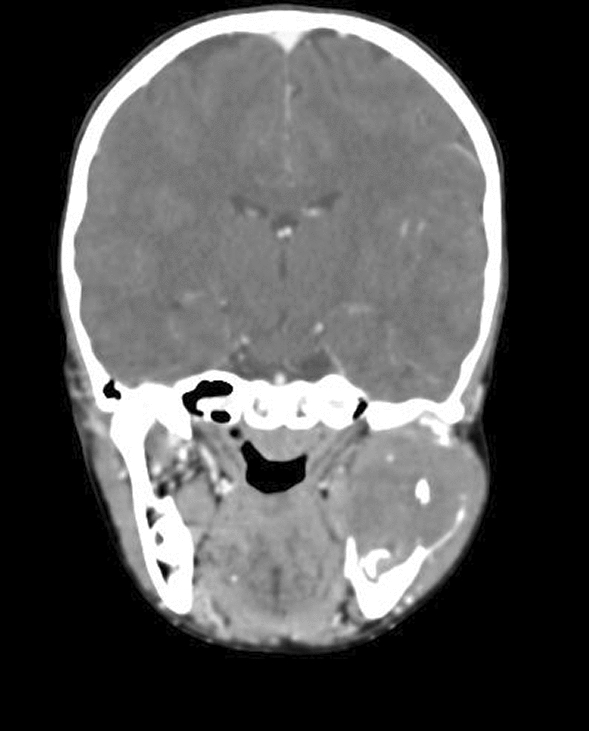
Coronal section of the contrast enhanced computed tomography scan showing gross destruction of the mandible

It was associated with pain, which was gradual in onset, dull aching, continuous, and localized in nature. The patient gave a history of trauma over the left posterior region of his face due to a fall from his bicycle 2 years ago. On examination, the extraoral swelling over the left side of the face, extending anteroposteriorly from the corner of the mouth to the posterior border of the mandible on the left side, and superoinferiorly from the root of the helix to the inferior border of the mandible on the left side, measured 6 cm × 5 cm in greatest dimensions with no cutaneous sinuses. The swelling was tender and firm on palpation. There was history of intraoral pus discharge for 1 month following an incisional biopsy, along with the placement of a rubber drain intraorally from the left side of the lower jaw, which was done at another centre. There was onset of gradual trismus following the biopsy. The mouth opening of the patient was 12 mm when he reported to us. There was evidence of inflammation posterior to the first molar on the left side of mandible. The biopsy report was suggestive of nodular fasciitis. A computed tomography (CT) scan (with contrast) of the head suggested evidence of a well-defined, expansile lytic lesion with a homogeneously enhancing soft tissue density component noted in the left angle, ramus, and condylar and coronoid processes of the left hemimandible involving the mandibular canal measuring 4.6 cm × 4.5 cm × 5 cm. The lesion had corticated boundaries and was causing erosion of the underlying bone with periosteal elevation. The lesion extended into the lateral pterygoid and temporalis muscles at its insertion (Figs. 2, 3).

**Fig. 4:**
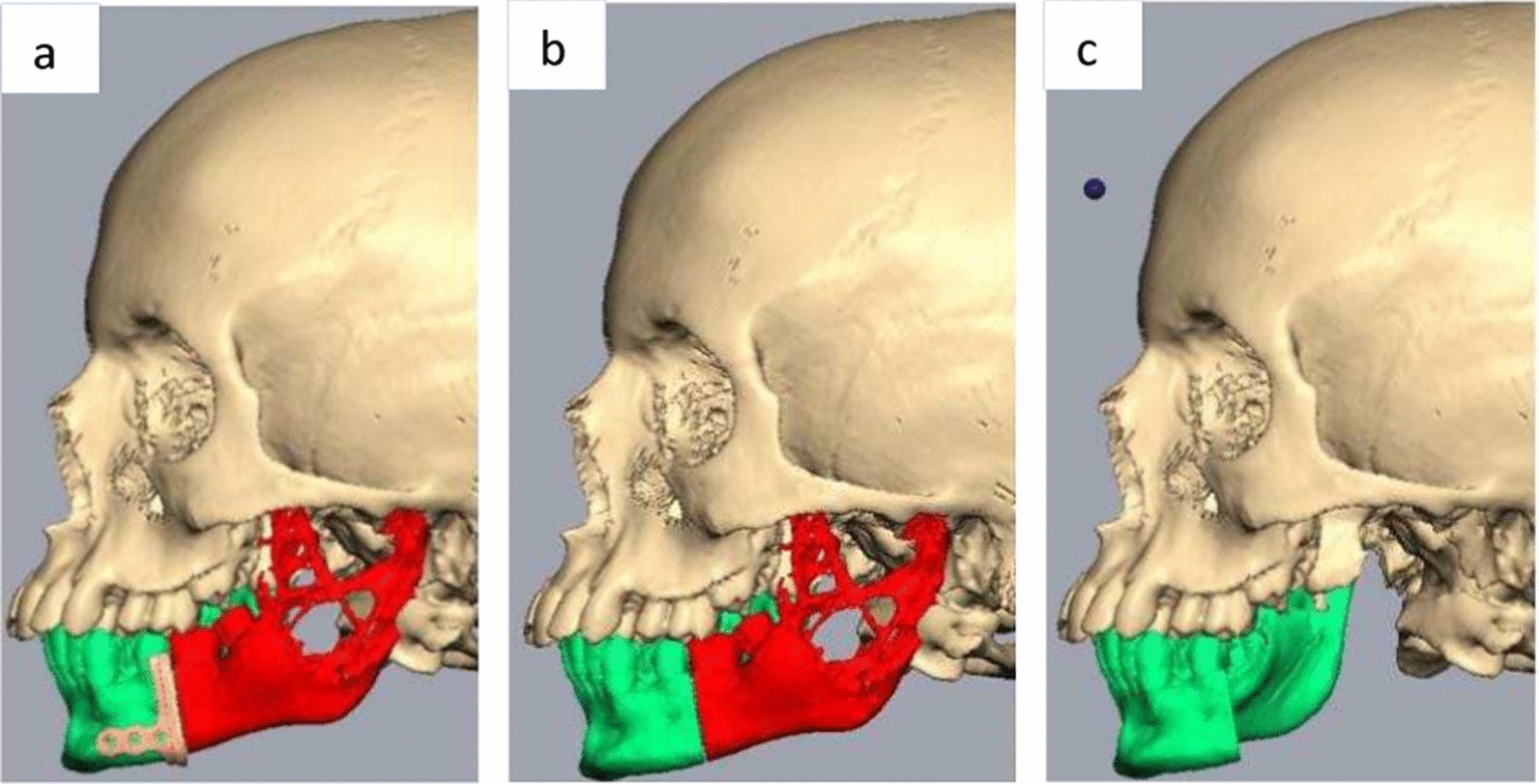
**a** Virtual surgical planning of the resection with placement of the cutting guide. **b** The red-colored portion depicts the amount of bone to be removed. **c** The green-colored portion depicts the amount of mandible preserved

Considering the extent of the lesion which had invaded into the mandibular bone involving the body, condylar and coronoid processes of the left side, the surgical management demanded removal of the diseased bone in its entirety. To rehabilitate the function and esthetics of the patient, reconstruction of the defect was planned using a fibula flap and a patient-specific glenoid fossa and condyle unit. The customized implant was designed and fabricated using Digital Imaging and Communications in Medicine (DICOM), which was sent to the manufacturer where 3D images were created using computer aided design and computer aided manufacturing (CAD/CAM) software. Virtual surgical planning began with a biomedical engineer from a modeling company and the operating surgeon 1 week preoperatively. The patient was also advised to undergo a computed tomography angiogram of the left leg, which was planned for fibula reconstruction. The virtual surgical planning included designing the resection margins, which was discussed by considering the computed tomography scan and the communication between the surgeon and the biomedical engineer (Fig. 4a–c).

**Fig. 5:**
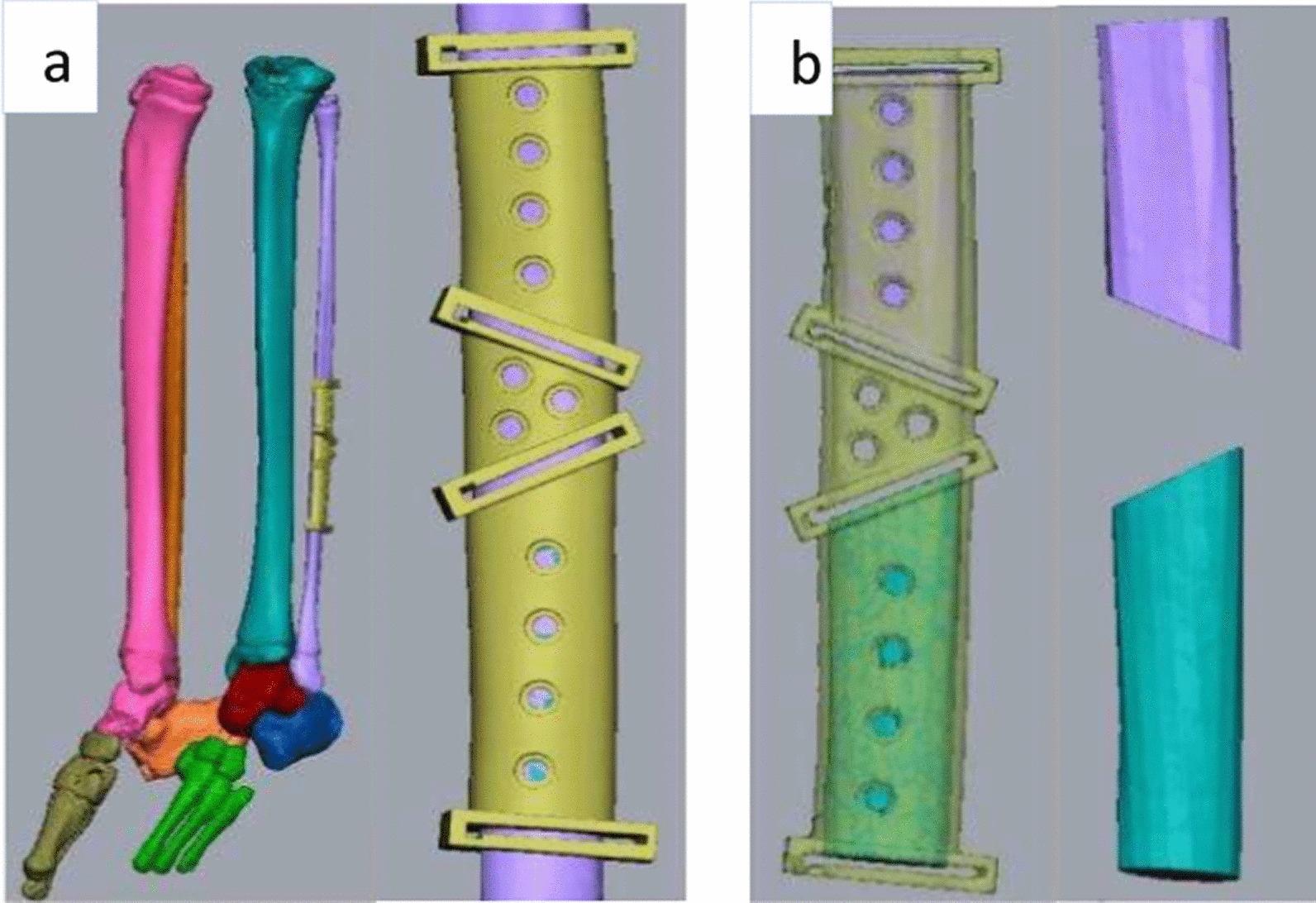
**a** Placement of the cutting guide on the fibula bone. **b** Osteotomized fibula bone using the cutting guides

The fibula cutting guides were fabricated on the basis of the design of the resection margin and by calculating dimensions of the bone from the CT angiogram (Fig. 5a, b).

**Fig. 6:**
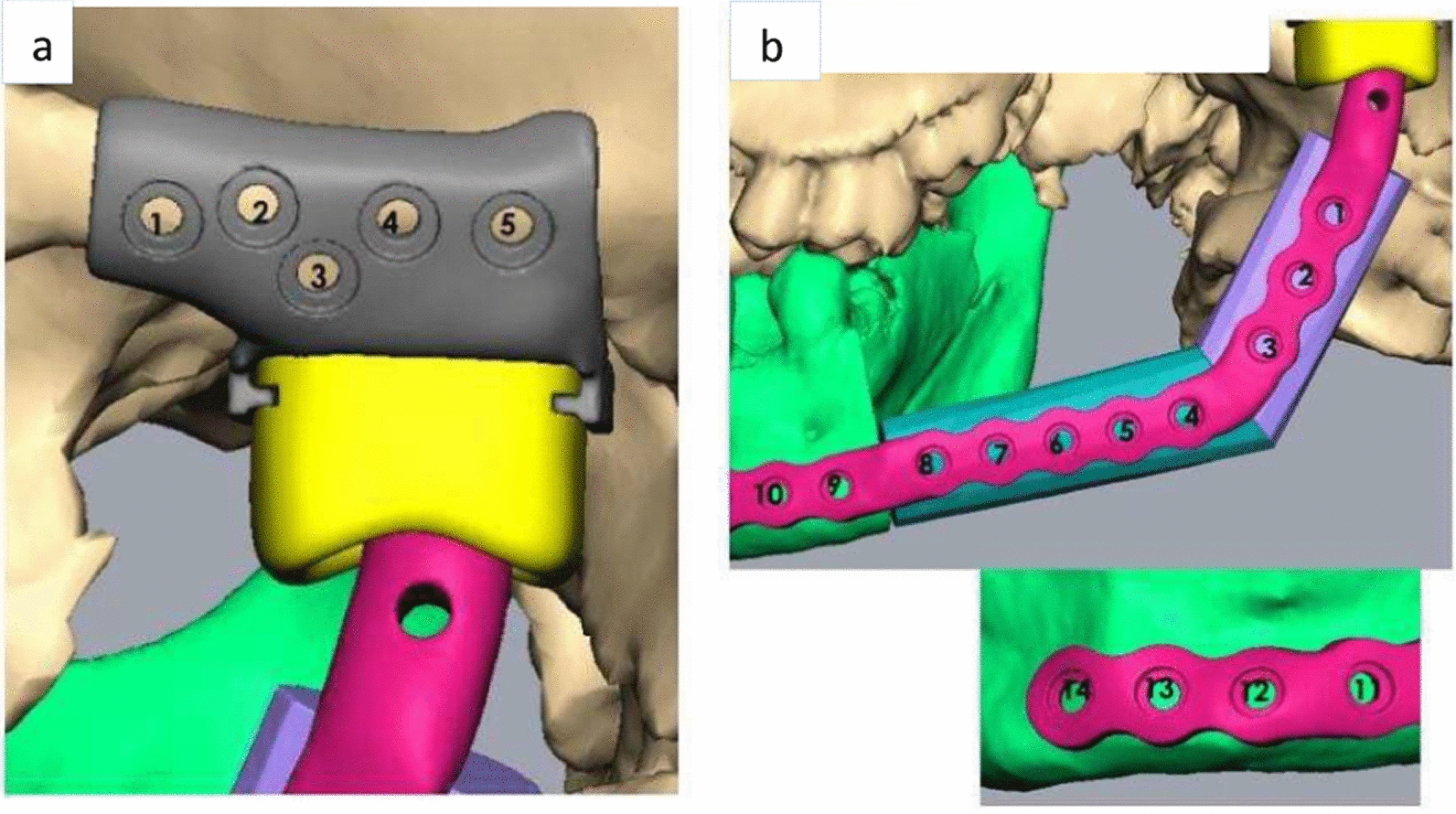
Virtual surgical planning of **a** glenoid fossa component along with screw placement, **b** recon plate over the fibula bone and the preserved mandible along with screw placement

The individual segment size prediction was also done with virtual surgical planning. A stereolithographic model and patient-specific condyle and glenoid fossa component was prepared on the basis of the planning (Fig. 6a, b). The implant was fabricated and sterilized prior to the use.

**Fig. 7:**
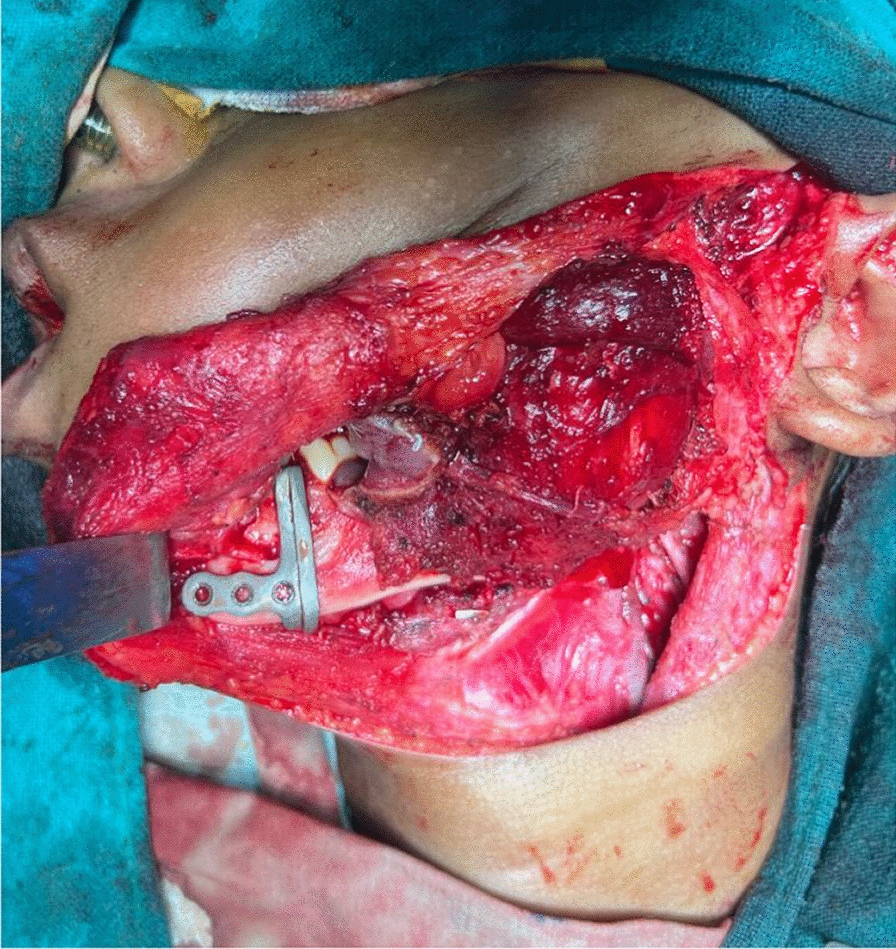
Resection using cutting guide

**Fig. 8:**
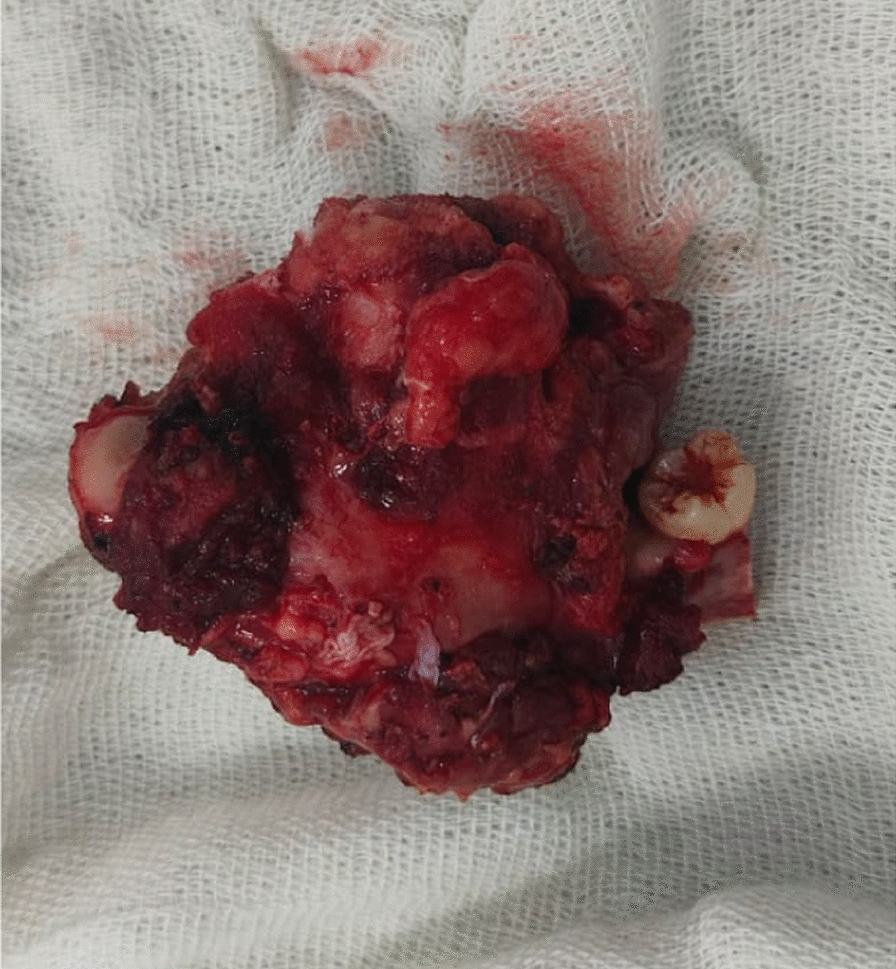
Resected specimen

Under general anesthesia, Apron’s incision was given over the left side of the neck and the expansile lesion was exposed. Wide local excision of the lesion from mucosa, buccinator muscle, masseter muscle, lateral pterygoid, and medial pterygoid muscle was done. Adapting the cutting guide over the mandible as per the surgical plan, segmental mandibulectomy from the second premolar to disarticulation of the condyle on the left side was carried out (Figs. 7, 8).

**Fig. 9:**
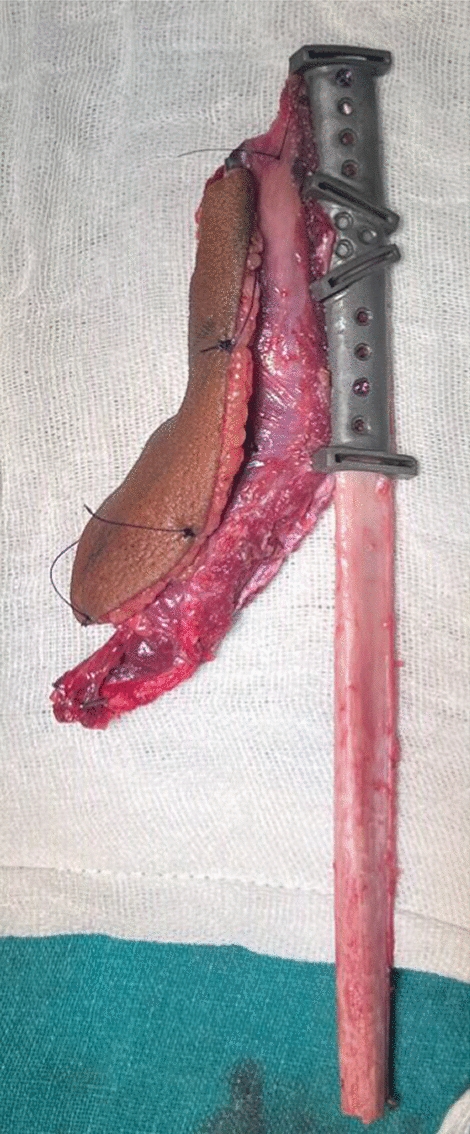
Using cutting guides to determine the osteotomy cuts

Taking the surgical defect into consideration, an osteomyocutaneous fibula flap, 28 cm in size, was harvested from the left leg. The osteotomy cuts for the adaptation of the fibula into the defect was done with the cutting guides, which were specific to the measurements of the patient’s mandible (Fig. 9).

**Fig. 10:**
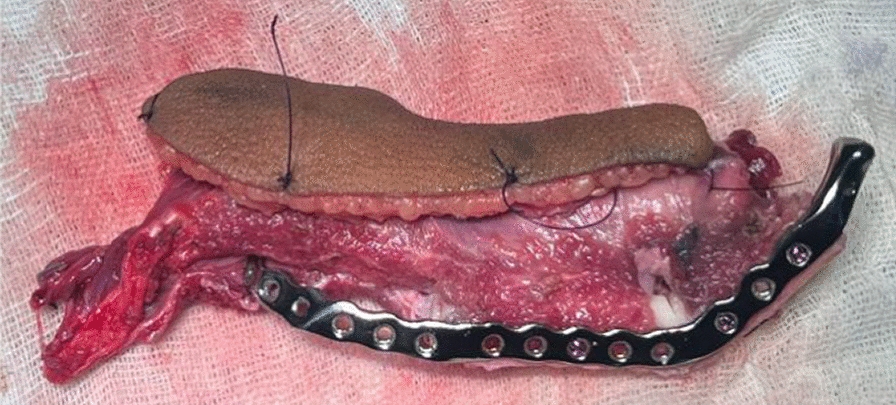
Osteotomized fibula fixed with the recon plate

**Fig. 11:**
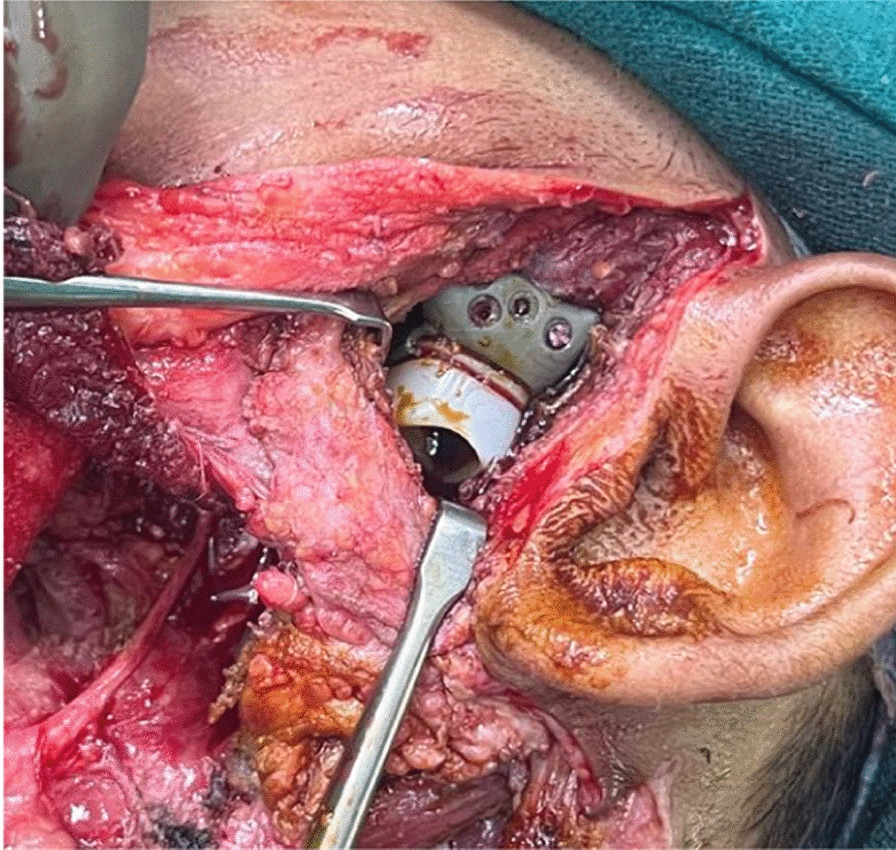
Fixation of the glenoid fossa component

The osteotomized segment of the fibula, which now forms the future ramus and body of the mandible were fixed together with the patient-specific recon plate (Fig. 10). Before setting the fibula flap into the defect, the patient-specific polyethylene terephthalate glenoid fossa component was fixed (Fig. 11).

The fibula flap was then adapted to the defect and was fixed to the cut end of the mandible with titanium screws. Anastomosis was performed with the facial artery and external jugular vein. The skin paddle was sutured intraorally into the defect. A negative suction drain was placed in the neck and left leg donor site. The neck and donor site were closed.

The patient was shifted to the pediatric intensive care unit without being extubated nor reversed. The patient was sedated till next morning to prevent neck movements due to agitation. The next day, the patient was reversed, extubated, and started on nasogastric tube feeds, and then moved to the ward for further monitoring. He was given intravenous antibiotics for 5 days. Postoperatively, there was mild edema on the left side of face. The negative suction drains were removed on the 5th day postoperatively and the patient was discharged.

Histopathologically, the resected specimen showed predominantly bland spindle cells arranged in fascicles and, at places, short bundles. There was minimal myxoid activity. The stroma showed predominantly collagenous matrix with few areas showing myxoid changes. The stroma also consisted of prominent small blood vessels, otherwise unremarkable, with scant scattered nonspecific inflammatory infiltrate. The immunohistochemistry findings were strongly positive for SMA and vimentin, and negative for S100 and CD34 markers. These features were suggestive of nodular fasciitis.

**Fig. 12:**
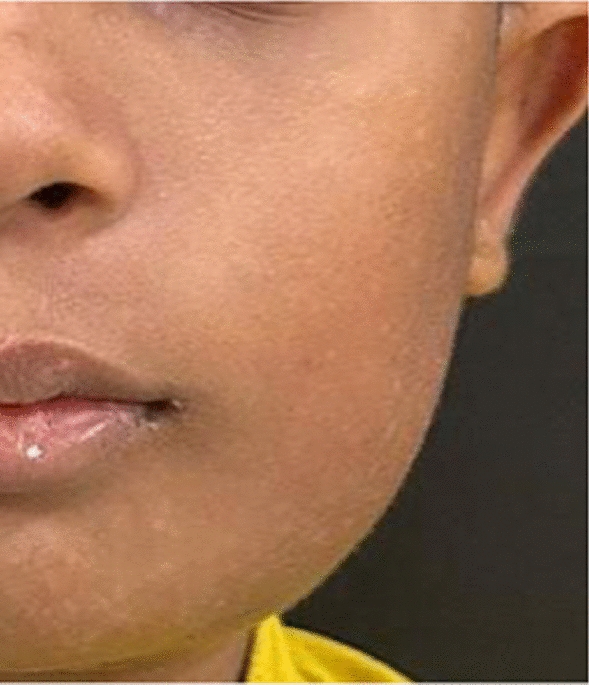
Frontal photo of the patient 3 months postoperatively

At 3 months postoperatively, the face had mild asymmetry (Fig. 12). However, the surgical site and the donor site had healed satisfactorily. There was no gait disturbance.

## Discussion

Nodular fasciitis is a benign, discrete proliferation of fibroblasts, which appears as a solid or cystic mass in the subcutaneous tissue. Its rapid growth resembles a malignant process, such as sarcoma. The commonly affected areas of the head and neck are the maxillofacial region, forehead, scalp, postauricular region, and the parotid [11].

Local excision of the lesion is considered the treatment of choice, particularly in cases where the lesion arises in the extremities and the trunk. The aim of the excision is to eliminate the lesion entirely, to facilitate accurate diagnosis through histopathological analysis, and to avoid any potential recurrence. The incidence of recurrence of the lesion after local excision has been reported to be 1–6% [5].

However, spontaneous regression of the lesion after biopsy of an early lesion has also been reported. Hence, observation was a common strategy used in small, non-painful, and asymptomatic growths. The monitoring of any potential growth or changes in the lesion would require further surgical intervention [12].

Nonsurgical methods, such as intralesional triamcinolone injections to reduce the size of the lesion, have been documented by Choi *et al*., which provided effective symptomatic relief and reduced inflammation in candidates with mild symptoms [13]. Oh *et al*. retrospectively analyzed the outcomes of surgical excision versus nonsurgical methods such as triamcinolone acetonide intralesional injections and the pinhole method with a carbon dioxide laser. Owing to the myxoid pattern of myofibroblast proliferation in nodular fasciitis, laser treatment proved effective in reducing the size of the lesion by promoting tissue contraction [14].

Anehosur *et al*. reported a case of a 10 year old boy, having a lesion of nodular fasciitis of size 7 cm × 5 cm × 5 cm extending from the alveolar region of the mandible superiorly and involving the cricoid region inferiorly. They performed an elective tracheostomy as there was a gross shift of the midline in the oropharyngeal region. They resected the tumor surrounding the left mandible and reinforced it with fixation with a stainless steel miniplate owing to extensive mandibular cortical erosion [15].

In our case, the lesion exhibited rapid progression over the course of just 4 months, raising significant concerns regarding the potential risk of airway obstruction. The lesion, a lytic mass, progressively eroded the hemimandible in its entirety, making segmental resection an inevitable course of action. The skin over the swelling was thin. Given the young age of the patient and the resulting segmental defect, reconstruction with bone was required to restore anatomical integrity. Since the condyle was resected, the resulting defect constituted a cantilever defect. Therefore, to prevent graft instability and ensure functional preservation, total joint replacement (TJR) was performed. Considering the patient’s age and ongoing growth phase, the surrounding soft tissue and bony structure development were expected to be appropriately guided by functional demands, as conceptualized by the functional matrix theory, ensuring optimal alignment and function.

## Conclusion

Although nodular fasciitis is a benign lesion, it has the potential to cause significant tissue damage. Prioritizing patient safety and well-being when selecting the appropriate treatment approach is essential. Personalized treatment strategies not only address the lesion but also support the patient’s functional rehabilitation. Regular follow-up is crucial to monitor for any signs of recurrence and ensure sustained recovery.

## Data Availability

The data utilized in the development of this manuscript are available upon request from the corresponding author.
